# Hybrid Inorganic-Organic Core-Shell Nanodrug Systems in Targeted Photodynamic Therapy of Cancer

**DOI:** 10.3390/pharmaceutics13111773

**Published:** 2021-10-23

**Authors:** Gauta Gold Matlou, Heidi Abrahamse

**Affiliations:** Laser Research Centre, Faculty of Health Sciences, University of Johannesburg, Doornfontein 2028, South Africa; goldm@uj.ac.za

**Keywords:** nanomedicine, targeted photodynamic therapy, core shell structure, hybrid nanodrugs, photosensitizers, colloidal drug-delivery systems

## Abstract

Hybrid inorganic-organic core-shell nanoparticles (CSNPs) are an emerging paradigm of nanodrug carriers in the targeted photodynamic therapy (TPDT) of cancer. Typically, metallic cores and organic polymer shells are used due to their submicron sizes and high surface to volume ratio of the metallic nanoparticles (NPs), combined with enhances solubility, stability, and absorption sites of the organic polymer shell. As such, the high loading capacity of therapeutic agents such as cancer specific ligands and photosensitizer (PS) agents is achieved with desired colloidal stability, drug circulation, and subcellular localization of the PS agents at the cancer site. This review highlights the synthesis methods, characterization techniques, and applications of hybrid inorganic-organic CSNPs as loading platforms of therapeutic agents for use in TPDT. In addition, cell death pathways and the mechanisms of action that hybrid inorganic-organic core-shell nanodrug systems follow in TPDT are also reviewed. Nanodrug systems with cancer specific properties are able to localize within the solid tumor through the enhanced permeability effect (EPR) and bind with affinity to receptors on the cancer cell surfaces, thus improving the efficacy of short-lived cytotoxic singlet oxygen. This ability by nanodrug systems together with their mechanism of action during cell death forms the core basis of this review and will be discussed with an overview of successful strategies that have been reported in the literature.

## 1. Introduction

Over the past few decades, research on the application of nanotechnology in medicine, science, engineering, and agriculture has grown tremendously due to the exciting and tunable properties of nanoparticles (NPs) [1]. Metallic NPs, such as silver, gold, copper, iron, silica, and titanium NPs, are attractive in nanomedicine due to their surface plasmonic resonance (SPR) properties and ability to be tuned with a change in size and shape [2,3,4]. Furthermore, properties of the NPs can be altered based on their composition, morphology, nature (organic or inorganic or polymeric), form (mono or bimetallic or hybrid), and their surface properties to meet desired functions [5,6,7].

Hybrid NPs represent an emerging class of NPs that are made-up of two or more components fused together, typically a metallic core and polymeric shell [8]. The choice of the core metal and polymer materials is based on the need for properties, functionality, and application [8]. Of interest to this review are hybrid inorganic-organic core-shell nanoparticles (CSNPs) for application in the targeted photodynamic therapy (TPDT) of cancer. Hybrid CSNPs consisting of an inorganic metal core and organic polymer shell offer synergistic properties in TPDT, i.e., the SPR properties of the core metal NPs that enhances the absorption of light in the therapeutic window (600–800 nm) [9,10], and the organic polymer shell enhances solubility, drug circulation, and colloidal stability of the nanodrug system [11,12,13,14]. Additionally, the polymer shell offers chemical functional groups to allow effective attachments of therapeutic agents, such as PS agents and cancer specific ligands [8,15].

TPDT follows the principle and mechanism of conventional photodynamic therapy (PDT). An administered PS agent is illuminated with light of specific wavelength to generate cytotoxic singlet oxygen that is responsible for causing the death of cancer cells [16]. TPDT evolves from the shortfalls of PDT, such as the poor solubility of PS agents in aqueous media and limited concentration of the PS agent at the tumor tissue [17,18,19]. TPDT takes advantage of nanotechnology to formulate PS agents attached to nanocarriers to achieve specific targeting, controlled drug release, and concentration of the PS agents in the solid tumor environment [20,21].

The PS agents attached to nanocarriers are then able to localize within the subcellular compartments of the solid tumor, enhancing the efficacy of the short-lived cytotoxic singlet oxygen [22,23,24,25]. Tumor selectivity of the PS agent is also improved when cancer specific ligands are incorporated in the nanodrug system [25]. NPs have sub-micron sizes and high surface to volume properties [26] that allow for the loading and delivery of therapeutic agents through passive and active targeting of the solid tumor. Nanodrug systems can easily deliver loaded therapeutic drugs through leaky blood vessels of the tumor tissue due to their submicron sizes, known as the enhanced permeability and retention (EPR) effect (passive targeting) [17,25,27,28]. Active targeting involves loading a cancer targeting ligand as part of the nanodrug system to improve the specificity and localization of the therapeutic drugs by binding to target receptors on or within the tumor tissue [17,25,27,28]. Thus, the development of nanodrug systems with biocompatibility and stability for the selective and specific targeting of cancer cells is crucial for in-vitro and in-vivo application.

This review highlights the synthesis and characterization of hybrid inorganic-organic CSNPs as loading platforms of therapeutic agents, such as PS agents and cancer specific ligands, and their use as delivery platforms in TPDT. Organic polymer shells play an important role as they offer absorption sites for the attachment of therapeutic agents [29]. Additionally, an organic polymer shell is able to enhance the colloidal stability, cytocompatibility, and bio-functionality of the hybrid nanodrug system [1,30]. Since the modality of PDT is dependent on the subcellular localization of the PS agents to optimize the efficacy of cytotoxic singlet oxygen or ROS through different cell death pathways [24,25], this review also highlights the specific target ligands and strategies that have been adopted to deliver and concentrate PS agents in the solid tumor, thus achieving an efficient PDT effect on cancer cells through the cytotoxic singlet oxygen killing of cancer cells [17].

## 2. Targeted Photodynamic Therapy

### 2.1. Photodynamic Therapy

**Figure 1:**
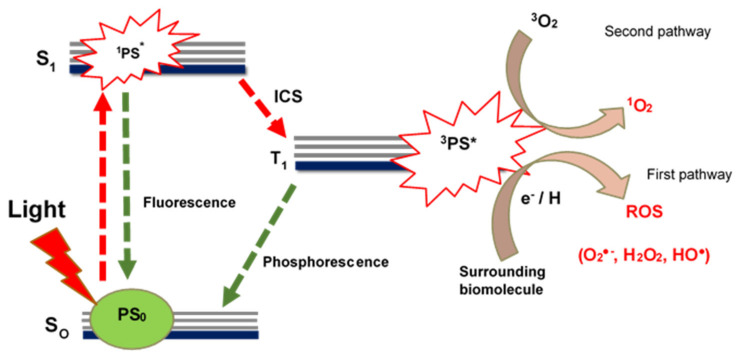
Schematic representation of the Jablonski diagram depicting the photochemical and photophysical processes that a PS agent undergoes during PDT after illumination with laser light in the body. S_0_ (singlet ground state), S_1_ (Singlet excited state), ICS (Intersystem crossing), T_1_ (Triplet excited state) and ROS (Reactive Oxygen Species). PS_0_ (PS in its ground state), ^1^PS* (PS in the singlet excited state) and ^3^PS* (PS in the triplet excited state).

Photodynamic therapy (PDT) is a clinically approved cancer treatment modality that is less invasive compared to surgery and chemotherapy [31,32]. PDT achieves its cancer killing efficacy by combining two non-toxic components, i.e., a photosensitizer (PS) agent and a light of specific wavelength to generate cytotoxic singlet oxygen that causes irreversible photodamage of cancer cells [33,34]. Figure 1 illustrates the photochemical and photophysical processes that a PS agent undergoes to generate reactive oxygen species (ROS) or cytotoxic singlet oxygen species that are responsible for photodamage on cancer cells.

Typically, a PS agent is administered and allowed to distribute and accumulate at or within the tumor tissue [22]. Once accumulation time is complete, the PS agent in its ground state (PS_0_) is illuminated with a light of specific wavelength (Figure 1). Upon illumination, the PS agent absorbs energy and transitions to the singlet excited state (S_1_). In the singlet excited state, the PS agent in the singlet excited state (^1^PS*) can either fluorescence back to the ground state or undergo intersystem crossing (ISC) into the excited triplet state, with the desirable process here being the ISC where the PS agent transitions into excited triplet state (T_1_) [21,22] (Figure 1). In the excited triplet state (^3^PS*), PS agents can follow two possible photochemical pathways in a biological environment that results in cancer photodamage during PDT, named Type I (first pathway) and Type II (second pathway) (Figure 1).

In the Type II (second) pathway, the PS in the excited triplet state transfers its energy to molecular oxygen (^3^O_2_) to result in a highly reactive and cytotoxic singlet oxygen (^1^O_2_) [35] (Figure 1). The interaction of the cytotoxic singlet oxygen with near-by biological molecules, such as nucleic acids, lipids, and proteins of the cell membrane, results in cell death through necrosis or apoptosis [35]. In the Type I (first) pathway, the PS in the excited triplet state interacts with surrounding tumor tissue biomolecules to acquire an electron or a hydrogen atom and generate ROS, such as peroxide radicals (HO●), superoxide anion (O_2_^●−^), hydroxyl ion (HO^−^), and hydrogen peroxide (H_2_O_2_) [22,35] (Figure 1). The ROS causes destruction of normal functions by damaging the cell membrane through lipid peroxidation [35]. Studies show that many PS agents typically follow the Type II pathway in PDT [18,35], although both pathways can occur at the same time while a balance between the pathways is only dependent on the PS and its affinity to tumor tissue and the amount of available oxygen [18].

### 2.2. Photosensitizer Agents

PS agents represent one of the important components in PDT, together with molecular oxygen and laser light [36]. The selection of an ideal PS agent is based on the factors that will improve the efficacy of PDT on cancer cells [37]. An ideal PS agent should have a high absorption coefficient in the 650–850 nm region, be non-toxic to cells in the absence of light, be highly soluble in aqueous media to easily permeate through biological barriers during distribution and accumulation, and finally be able to generate high cytotoxic singlet oxygen or ROS [36,37]. One of the major advantages of PDT is that the non-toxic PS agent can be used as a therapeutic drug in combined therapy with other modalities, such as chemotherapy, immunotherapy, radiotherapy, and photothermal therapy [31].

PS agents are typically developed based on the porphyrin or non-porphyrin PS agent chemical classes [3,31,38]. The first-generation PS agents such as hematoporphyrin derivatives (HpD) (porphyrin monomers, dimers and oligomer) were developed from porphyrin backbone in the 1970–1980′s [3]. HpD were the earliest PS agents to reach clinical trials under trade name Photofrin [31,38]. Their weak absorption in the red and far-red region (625–750 nm) where optimal tissue penetration of light can be achieved has thus been their biggest limitation [3]. Second-generation PS agents were then developed to overcome the shortcomings of the first-generation PS agents, these complexes includes phthalocyanines, chlorins, pheophorbides, texaphyrins, xanthenes, and phenothiazines [39]. They have high extinction coefficients, high singlet oxygen generating abilities, are able to absorb light at wavelengths longer than 630 nm, and exhibit greater cytotoxicity on tumor tissue compared to HpD [3,40].

A limitation in the PDT efficacy of second-generation PS agents results from the reduced photosensitizing efficiency that is caused by their hydrophobicity and aggregation in water [3,19,40]. Furthermore, their photoactivity is only strongest in their monomeric forms, thus minimizing their ability to generate high yields of cytotoxic singlet oxygen [19]. PS agents could also poorly distribute or localize at the tumor tissue, thus minimizing the efficacy of PDT on cancer cells [25]. Since the cytotoxic singlet oxygen or ROS agents have a limited lifetime and radius in biological systems, a lack of specific localization, or subcellular accumulation of the PS agents in tumor tissue, PDT efficacy is therefore hindered [19,24,25]. This makes the efficacy of PDT heavily dependent on the subcellular localization or accumulation of the PS agent within the cancer cells or tissue [24,25].

Recently, PS agents have been developed with cancer targeting specificity to overcome limitations of conventional PS agents and improve the efficiency of the cytotoxic singlet oxygen on cancer cells [41]. As such, new PS agents with tumor specific targeting has led to the modality of targeted PDT (TPDT) [17,41]. In TPDT, two common approaches are used to improve tumor targeting specificity of PS agents, the first approach being the conjugation of PS agents to cancer specific biomolecules to improve accumulation of PS agents at the tumor tissue through active targeting [17], and the second approach takes advantage of the leaky vasculature system around tumor tissue to deliver PS agents through passive targeting when conjugated to nanocarriers such as CSNPs [17,42].

### 2.3. Active Targeting of TPDT

**Figure 2:**
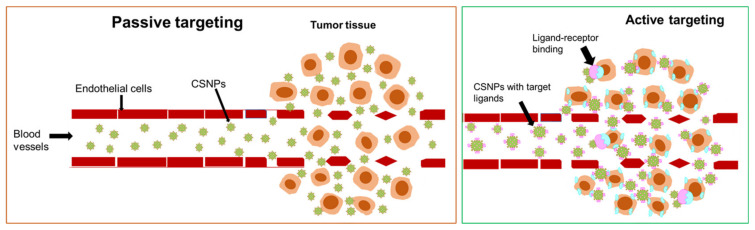
Illustration of drug delivery mechanisms followed by hybrid nanodrug systems through active and passive targeted photodynamic therapy.

Active targeting is a tumor targeting strategy that develops new generation PS agents coupled to cancer specific biomolecules to improve the subcellular accumulation of PS agents within tumor cells or tissue [43]. Tumor growth is associated with the overexpression of certain receptor molecules that serve as nutrients during proliferation stages [17]. Typically, PS agents or nanocarrier systems can be loaded with ligands or antibodies that can attach or bind with great affinity to receptors or antigens overexpressed on the tumor surface, Figure 2. This method not only achieves the accumulation of PS agents at the target site, but also improves endocytosis of the therapeutic agents to maximize PDT efficacy [43,44].

Ligands, such as aptamers, peptides, folic acid, proteins, and antibodies, have been extensively researched for active targeting in TPDT of cancer [17,25,44,45]. CSNPs have desirable surface properties that can be utilized for loading an array of chemical and biochemical molecules that includes PS agents and cancer specific ligands [5,46] (Figure 2). In 2012, Benachour et al. developed a multifunctional CSNPs drug delivery system consisting of a gadolinium oxide core and silica shell functionalized with a chlorin based PS agent and a peptide for the active targeting of neuropilin-1 (NRP-1) overexpressed in tumor angiogenic vessels [47]. The CSNPs drug system was demonstrated to bind effectively to the NRP-1 ligand and remain able to retain the photosensitizing properties and cytotoxicity of the PS agent on cancer cells (MDA-MB-231) [47].

### 2.4. Passive Targeting of TPDT

In passive targeting of tumor cells or tissue, the irregular tissue architecture of solid tumor environment is typically taken advantage of to deliver therapeutic drugs to the tumor tissue [25]. The rapid tumor proliferations cause blood vessels around the tumor tissue to develop extensive angiogenesis, vascular permeability, and a weakened lymphatic system [5,25] (Figure 2). Thus, the passive targeting of tumor tissue essentially requires that PS agents be loaded on a carrier system of sizes small enough to permeate and accumulate at the tumor tissue through leaky vascular system of the tumor, yet large enough to avoid leaking back into blood capillaries [48,49] (Figure 2). Such a phenomenon is known as the enhanced permeability and retention (EPR) effect and forms the basis of passive targeting of the tumor [50].

CSNPs have submicron sizes, easily modifiable surfaces, and multifunctionality that is based on design, size, and shape, making them attractive to use as drug carriers for passive targeting [1,51] (Figure 2). These properties allows CSNPs to easily load therapeutic drugs, breach through physiological barriers, and effectively deliver loaded drugs with great internalization and high accumulation at the disease site [5,46]. Liu and co-workers in 2014 synthesized a series of inorganic-organic CSNPs consisting of Au, Ag, Cu, Fe_3_O_4_, and TIO_2_ core with poly (styrene-*alt*-maleic acid) (PSMA) shell [29]. The PSMA shell consists of polystyrene blocks, which allows for the attachment of aromatically structured therapeutic agents, such as doxorubicin, chlorin e6, methylene blue, and many others. In this study, methylene blue (MB) was loaded on the Au@PSMA surface through π-π stacking attachment and applied to PDT where the Au@PSMA promoted passive targeting of the nanodrug system on cancerous cells, leading to improved efficacy of the ROS on cancer cells [29].

## 3. Inorganic-Organic CSNPs

### 3.1. Background on Inorganic-Organic CSNPs

CSNPs are a class of hybrid nanoparticles that consists of two inorganic metals fused together or an inorganic metal blended with an organic or two organic materials making up the core and shell of the nanoparticles [52]. This leads to four classifications of CSNPs, inorganic-inorganic CSNPs, inorganic-organic CSNPs, organic-inorganic, and organic-organic CSNPs [1]. Inorganic-organic CSNPs are very attractive in biological applications due to the nature of the core and the shell material. Inorganic metals are commonly used as core metals while biocompatible organic polymers are used as shells. Inorganic metals, such as Au, Ag, and Fe, are readily available and non-toxic with unique properties that can be exploited to adopt any modification [1,52]. The surface plasmonic resonance (SPR) frequency of these metals is also an important property in TPDT as it allows for a spectral region or wavelength of interest in the visible to near-infrared region to be achieved with desired size and shape during synthesis [52].

The introduction of an organic shell or polymer coating is crucial for colloidal stability in suspension media and functionalization sites for the attachment of therapeutic drugs or cancer specific ligands for active targeting [19,53]. Organic shells or coatings are also advantageous as they can offer electrostatic and steric forces that enhance colloidal stability and prevent the aggregation of nanoparticles during application [54,55]. Thus, a uniform and suitable organic polymer choice is important for a biologically viable inorganic-organic CSNPs system [52]. The resulting hybrid inorganic-organic CSNPs have major advantages in biological applications such as TPDT. In addition to nano-sizes, the CSNPs are also able to offer better conjugation to therapeutic drugs and cancer targeting ligands with increased cyto-compatibility and dispersion [56].

### 3.2. Synthesis of Inorganic-Organic CSNPs

**Figure 3:**
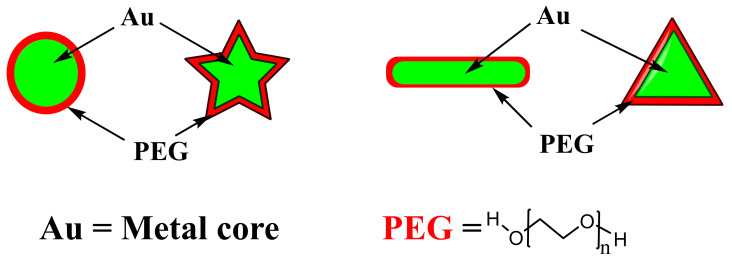
Typical hybrid inorganic-organic CSNPs made of differently shaped gold core and PEG shell, differently shaped AuNPs are achieved during the synthetic procedure [52].

The synthesis of inorganic-organic CSNPS with biological properties, such as compatibility and colloidal stability, can be achieved with various methods, as listed in Table 1. Various methods have been used successfully to synthesize CSNPs with a metallic core and organic shell, including chemical reduction, thermal decomposition, sol gel method, and the sequential or step by step method [52,57]. The complete synthesis of inorganic-organic CSNPs typically follows a two-step approach, starting with the synthesis of the inorganic metal core followed by coating or the addition of the biocompatible organic shell, usually a polymer component [19,53]. A stable and uniform thickness of the shell is critical as a larger shell size can impact the properties of the core metal [52]. Figure 3 illustrates a typical inorganic-organic CSNPs structure synthesized under different reaction conditions, thus with different shapes of the core [52]. The polymer size, attachment of therapeutic agents, and subsequent shell size can also significantly increase the size of the overall CSNPs to above 200 nm, a non-ideal size for EPR targeting.

Chemical reduction is a facile method that is commonly used to develop metal NPs or the metallic core of CSNPs due to its simplicity and easy control over the size and shape of the core. Xiang et al. used a chemical reduction method to synthesize a bimetallic core component consisting of Au and Pd (AuPd), which was followed by coating with a biocompatible polymer shell (poly pyrrolidone (PvP)) in the presence of a reducing agent [58]. The resulting AuPd@PvP CSNPs was produced with a suitable size and morphology that demonstrated excellent therapeutic effect on cancer cells [58]. The sol-gel method is also a simple method that can be used to prepare metal core of the hybrid CSNPs. This method is more efficient in liquid-solid transformation for metal oxide cores [52]. Wang et al. used the sol-gel method to develop an inorganic-organic CSNPs consisting of a multicore metal of Au and SiO_2_ coated with poly pyrrole (PPy) [59]. The hollow inorganic nanostructured Au@SiO_2_@PPy have excellent properties, such as easy functionalization and good flowability, to allow for use in therapeutic applications [59].

The one-pot synthesis method is another interesting method that has been used to successfully synthesize a series of inorganic-organic CSNPs by Liu et al. [29]. Au, Ag, Cu, Fe_3_O_4_, and TiO_2_ were used as inorganic metallic core components of the CSNPs, while the poly(styrene-alt-maleic acid) was used as an organic shell of the CSNPs [29]. The authors were able to achieve in-situ control over the size and shape of the CSNPs with a stable polymer encapsulation of the metallic cores [29]. Other methods that have been successfully used to synthesis inorganic-organic CSNPs are listed in Table 1 with relevant examples, including the thermal decomposition, seed-mediated synthesis, and emulsion methods. These methods are able to produce CSNPS with the right size and shape of the metallic core and the uniform and stable organic polymer shell with desired bio-functionality for therapeutic drug loading.

### 3.3. Characterization of Inorganic-Organic CSNPs

The successful preparation of inorganic-organic CSNPs can be established by characterizing the CSNPs with different microscopic, spectroscopic, light scattering, and thermogravimetric techniques. Such techniques are used to study and identify the CSNPs composition, morphology, crystal structure, chemical bonding, and composition as well as their electronic transitions [52]. The attachment of therapeutic agents, such as PS agents and cancer specific ligands, on the surface of CSNPs depends heavily on the chemical functionality of the polymer shell. Thus, the identification of chemical functional groups and bonding of elements using X-ray photoelectron spectroscopy (XPS) and Fourier transform infrared spectroscopy (FTIR) is crucial to prove the successful formulation of the inorganic-organic CSNPs with therapeutic agents attached [52,60]. XPS presents even greater benefits of identifying electronic states or binding modes of surface ligands, empirical formulas, depth analysis, and chemical status of the therapeutic agents attached to the inorganic-organic CSNPS surface [52,61].

Ultraviolet and visible (UV-VIS) light spectroscopy can be used to study the absorption properties (electronic transitions) of CSNPs when alone or loaded with therapeutic drugs. The characterization of CSNPs with UV-VIS and dynamic light scattering (DLS) coupled with zeta potential spectroscopy for therapeutic applications is particularly important for identifying the absorption spectra and colloidal stability of the CSNPs loaded with therapeutic agents, respectively. DLS is also able to determine the hydrodynamic size and surface charge of the CSNPs while alone or loaded with therapeutic agents for TPDT [52,62]. Colloidal stability testing of nanocarriers using DLS can be achieved in supplementary cell culture media, water, and phosphate buffer saline [62]. Further studies of the interaction of the nanocarriers (inorganic-organic CSNPs) with proteins can be achieved by using differential centrifugal sedimentation (DCS) [62].

Transmission electron microscopy and scanning electron microscopy are the two most common microscopic techniques that are used for the imaging of nanomaterials and to identify the size and shape of nanoparticles [1]. At high resolution, the two techniques are also able to distinguish the contrast of the metallic core and the organic polymer shell of the inorganic-organic CSNPs, thus assisting in determining the shell thickness (size) and core size of the CSNPs. Powder X-ray diffraction spectroscopy is also an extensively used technique for identification of the crystal structure and amorphous structures of the nanomaterials [52]. XRD is an important tool as it is able to identify the lattice structure and phase of the metallic core of the CSNPs and the amorphous peaks of the bio-polymer shell and attached therapeutic agents [60], thus confirming the development of the inorganic-organic CSNPs loaded with therapeutic agents. Since TPDT involves laser administration, the thermal stability of the CSNPs can be tested with thermogravimetric analysis (TGA). TGA is able to measure the degradation of materials through monitoring mass loss over time when exposed to high temperatures [63].

**Table 1:** Synthetic methods and characterization techniques of hybrid inorganic-organic CSNPs as reported in literature.

Inorganic Core	Organic Shell	Synthetic Method	Characterization Techniques	Ref.
Au, Ag, Cu, Fe_3_O_4_, TiO_2_	Poly (styrene-*alt*-maleic acid) (PSMA)	One pot synthesis	TEM, UV-VIS and XRD	[29]
AuPd	Poly (vinyl pyrrolidone) (PVP)	Chemical reduction	TEM, SEM, UV-VIS-NIR	[58]
Ag-Au	Poly (ethylene-glycol)-Hyaluronic acid (PEG-HA) hybrid	Reduction and Precipitation polymerization	TEM, UV-VIS, DLS, and PL	[64]
Carbon quantum dots (CQD)	Poly-l-lysine (PLL)	Thermal decomposition	FTIR, TEM and DLS	[65]
AgCl	Poly (aniline (PANI)	Reduction	SEM	[66]
Au-Ag	PEG	Seed mediated synthesis	TEM, UV-VIS and DLS	[67]
Ag-SIO_2_	Poly (pyrrole) (PPy)	Sol-gel method	TEM, XRD, RAMAN and TGA	[59]
Au	Poly (*DL*-lactic-co-glycolic acid) (PLGA)	In-situ reduction method	SEM, TEM and UV-VIS	[68]
Fe_2_O_3_	PEG and PLL	Thermal decomposition	TEM, FTIR, XPS	[69]
Fe_2_O_3_	PLGA	Emulsion and solvent evaporation method	DLS, TEM and SEM	[70]
SiNP	Alginic acid (ALG)	Step-by-step method	DLS, TEM	[71]
Au	PEG	Reduction	TEM, DLS, XRD	[72]

## 4. Inorganic-Organic CSNPs Drug Systems in TPDT

Inorganic-organic CSNPs have diverse properties that allow them to function as sole ROS producing agents (PS) or nanocarriers of conventional first- and second-generation PS agents in TPDT. The choice of the core metal, such as Au, Ag, and Fe, with SPR properties allows the CSNPs to generate cytotoxic singlet oxygen or ROS when illuminated with light [73]. The mode of action involves the interaction with the mitochondria, nicotinamide adenine dinucleotide phosphate (NADPH) oxidase, and penetration through the membrane by their physiological properties (size, SPR, and surface chemistry) [73,74]. This leads to the production of ROS, which consequently results in cell membrane destruction, DNA damage, cell cycle arrest, and alterations in apoptosis [73,75].

Kuo et al. compared the PDT effect of gold nanorod core coated with poly styrene-*alt*-maleic acid shell (AuNR@PSMA) with AuNR@PSMA attached to indocyanine green (ICG) photosensitizer on lung cancer (A549) and human keratinocyte cells (HaCaT) [76]. The AuNR@PSMA generated significant singlet oxygen species to cause cancer killing alone, which improved upon the conjugation of the ICG PS [76]. Nadhman et al. demonstrated the PDT effect of silver doped zinc oxide (Ag@ZnO) core nanoparticles coated with PEG shell on Leishmaniasis [77], an infectious disease associated with skin cancer [78]. The inorganic-organic CSNPs generated ROS upon light excitation with sunlight, which easily caused the killing of *Leishmania* parasites. The PEG polymer molecule shells were demonstrated to improve the stability and bio-functionality of the Ag@ZnO core [77].

**Figure 4:**
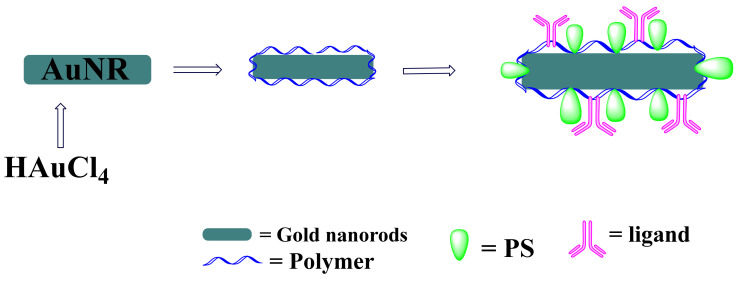
Schematic representation of hybrid CSNPs loaded with PS agents and cancer specific ligands for targeting and destruction of solid tumor through TPDT.

Attachments of PS agents on the surface of nanoparticles such as CSNPs are considered a “hot topic” in the treatment of cancer through TPDT [37]. Table 2 list examples of hybrid inorganic-organic CSNPs drug systems and their target mechanisms in TPDT of cancer. Both active and passive targeting TPDT can be achieved by taking advantage of the unique properties of specific size, shape, and surface functionality of CSNPs to attach cancer specific ligands together with PS agents [79]. Figure 4 presents an illustration of inorganic-organic CSNPs loaded with cancer specific ligands and PS agents. This was demonstrated by Kuo and co-workers by conjugating Anti-EGFR (epidermal growth factor receptor) monoclonal antibody on the surface of AuNR@PSMA and AuNR@PSMA-ICG to facilitate active binding on EGFR as overexpressed on A549 cancer cells, thus improving the accumulation of the inorganic-organic CSNPs drug system on the tumor surface [76].

Folic-folate receptor targeting is also one of the cancer specific targeting strategies commonly undertaken by many researchers in nanomedicine and drug delivery due to the overexpression of folate receptors on the surface of tumor cells [80]. Ghaznavi and co-workers developed an inorganic-organic CSNPs consisting of an iron oxide and gold core with a PEG functionalized folic acid shell (Fe_2_O_3_@Au-PEG-FA) to target specific binding to folate receptors expressed on tumor cells. The cytotoxicity of the Fe_2_O_3_@Au-PEG-FA nanodrug system was observed to be higher on human nasoharyngial carcinoma cells (KB) that present with high expression of folate receptors as compared to human breast adenocarcinoma cells (MCF-7), with low levels of expression of folate receptors on their membranes [81], indicating the significance of targeting ligands in efficient drug delivery and therapy. Apoptosis was found to be an underlying mechanism of cell death in this study [81].

The ability of nanodrugs or PS agents to concentrate within the cytoplasm of tumor cells is a crucial aspect in TPDT and the efficacy of the treatment. Using inorganic-organic CSNPs linked to therapeutic agents in PDT offers advantages of selective penetration, specific localization, and concentration at the tumor tissue, thus improving the efficacy of the ROS or short-lived cytotoxic singlet oxygen. Moreover, inorganic-organic CSNPs may offer protection of therapeutic agents from enzymatic degradation during biological treatment [36,82,83,84]. Generation of the cytotoxic singlet oxygen from PS agents is also known to improve when metal nanoparticles are linked to PS agents due to heavy atom effect which encourages ISC to the triplet state [82]. Shi et al. observed a higher PDT activity of the inorganic-organic CSNPs drug system (PEGylated iron oxide core and fullerene shell conjugated to hematoporphyrin monomethyl ether (Fe_2_O_3_@C_60_-PEG/HMME) on B16-F10 cancer cells and in-vivo on murine tumor model as compared to the PS agent (HMME) alone [85]. In addition to superior PDT efficacy, the magnetic properties of the Fe_2_O_3_@C_60_-PEG/HMME also allowed the hybrid nanodrug system to present excellent magnetic targeting and magnetic resonance imaging (MRI) properties, offering potential application as a multifunctional platform in cancer theranostic [85].

In another study, higher PDT activities (greater ROS yield) were observed by Liu and co-workers with a hybrid nanodrug system composed of multicore gold nanorods and mesoporous silica with a PEG shell which was functionalized with chlorin e6 and D-type cell penetrating peptide (AuNR@SiO_2_ (PEG)-d-CPP-Ce6) when tested on breast cancer cells, as compared to the PS agent (Ce6) alone [86]. In this study, active targeting of the lipids on cancer cell membranes was accomplished using D-type peptide. This improved the subcellular accumulation of the hybrid nanodrug system within the tumor tissue when tested on cancer bearing mice [86]. Additionally, the hybrid nanodrug system demonstrated both photothermal effect and PDT effect [86]. Similarly, specific localization of PS agents at the cancer site was achieved by incorporating anti-HER2 antibodies on hybrid nanodrug system (Au@PEG-ZnPc/anti-HER2) to target specific binding to the HER2 receptors on the cell surface [46]. The hybrid nanodrug system herein showed excellent cellular uptake and PDT efficacy against cancer cells with suitable stability in a biological environment and under irradiation with light [46]. 

## 5. Cell Death Pathways of Inorganic-Organic CSNPs Drug Systems in TPDT

PS agents in PDT commonly follows one or more of the three different pathways to cause death on cancer cells, namely necrosis, apoptosis, and autophagy [87]. Typically, the response of PDT on the cell death pathway may vary depending on the cell type, the PS agent, its subcellular localization and total fluence delivered [35,88]. However, the main instigator in PDT is the cytotoxic singlet oxygen that is responsible for initiating reactions and the activation of cell death pathways [35,89]. Nanocarriers such as hybrid inorganic-organic CSNPs are able to load, deliver and localize therapeutic agents such as PS agents and other cancer specific ligands in one or more cellular organelles that includes mitochondria, lysosomes, cell membranes and endoplasmic reticulum [35]. The direct pathways and mechanism of action followed by different hybrid inorganic-organic nanodrugs in TPDT are detailed below and summarized in Table 2.

### 5.1. Apoptosis

Apoptosis is a type of cell death that occurs under physiological conditions [35]. Several morphological changes occur, such as cell shrinkage, chromatin reduction, nuclear condensation, plasma membrane blebbing, and rounding up of the cell [90]. The mechanism of apoptosis follows an activation of a set of enzymes known as cysteine aspartate-specific proteases (caspases) and endonucleases [91,92]. This then leads to three distinct apoptotic pathways involving cell receptors, mitochondria, or endoplasmic reticulum (ER) [90,91,92]. In PDT, reactions that lead to the activation of apoptotic pathways are initiated by cytotoxic singlet oxygen or ROS molecules after irradiation [35]. 

Subcellular or intracellular localization of PS agents through active TPDT within cell organelles, such as mitochondria and lysosomes, is able to afford direct being of the lysosomal membrane or mitochondria, leading to proteases and cathepsins been released into the cytosol [93]. Liu et al. [86] studied the cell death pathways that the hybrid nanodrug system (AuNR@SiO_2_ (PEG)-D-CPP-Ce6) follows on MCF-7 breast cancer cells. The study found that the nanodrug system causes cell death through an apoptotic pathway with the mechanism that follows the activation of the death receptor enzymes (initiator caspase 8 and effector caspase 3/7), the opening of mitochondrial permeability transition pored and ROS generation [86]. An increase in caspase 3/7 activity was also observed to be a response to apoptotic pathway by Stuchinskaya et al. [46] using Au@PEG-ZnPc/anti-HER2 as a hybrid nanodrug system on breast carcinoma cell lines (SK-BR-3, MDA-MB-231, ATCC, and MCF-10A) [46].

### 5.2. Necrosis

Necrosis is characterized as an aggressive and speedy form of cell death that results from cell membrane loss due to excessive cell injury, caused by chemical or physical damage [35,90]. The outcomes of necrosis include cytoplasmic expansion, plasma membrane rupture, and the disruption of the organelles which leads to the uncontrolled release of intracellular contents and inflammation [90,94]. The biochemical mechanism of necrosis is typically classified in negative terms by the lack of caspase initiation, cytochrome c release, and DNA oligonucleosomal fragmentation [94].

During PDT, a high dose of PDT characterized as a high concentration of PS agents localized at a target tissue, a high irradiation, or both, and this is likely to cause cell death by necrosis due to the strong generation of the ROS or cytotoxic singlet oxygen [35,95]. The cytotoxic singlet oxygen or ROS molecules cause chemical damage to the cell membrane, leading to irreversible cell injury, and thus necrosis [95]. Liu et al. [96] found the cell death pathway of a hybrid nanodrug system (Fe_3_O_4_mSiO_2_@lipid-PEG-ZnPc-methotrexante) nanoparticle to be necrotic on cervical cancer (Hela) and lung cancer (A549) cells through excessive ROS induced membrane oxidation and perturbation [96]. In another study, necrosis was also found to be a cell death pathway when PEG-PEI@SIO_2_/ZnPc was used as a hybrid nanodrug system on mouse ascitic hepatoma cell line (H22) [97]. Lysosomal and cytosol disruption by the hybrid nanodrug system was found to be the mechanism that the nanodrug system followed to cause necrotic death on cancer cells by authors [97].

### 5.3. Autophagy

Unlike necrosis and apoptosis, autophagy is a catabolic cellular mechanism that strives to maintain a balance between the synthesis, degradation, and recycling of cellular products [35]. Autophagic processes mainly involve lysosomal degradation of the cellular organelles and proteins [35,94]. The mechanism of autophagy follows that an autophagosome (double membrane) that is surrounding a target region sequesters components of the cytoplasm including organelles and transports them to the lysosome to form an autophagosome-lysosome, the fusion which is then degraded by the lysosome hydrolase [35,94]

In PDT, autophagy can result in both cell death or cell survival, depending on the amount of cytotoxic singlet oxygen produced during the modality [98]. Autophagy achieves this by recycling the injured mitochondria or the endoplasmic reticulum before apoptosis can be initiated. In contrast, optimal PDT can result in oxidative stress stimulation of the lysosomal pathway, leading to the degradation of cytoplasmic contents [98]. In addition, localization of the PS agents within the lysosome compartment and its subsequent photodamage can potentially disturb the autophagic progress through inadequate clearance of the autophagic load [99]. Kim et al. [100] observed both necrosis and autophagy cell death pathways on Hela cells when using Au@GON-PEG-ZnPc as hybrid inorganic-organic nanodrug system. The nanodrug system was found to produce excessive amounts of ROS that resulted in oxidative stress on cancer cells, stimulating the lysosomal pathway of autophagy [100].

**Table 2:** Examples of inorganic-organic CSNPs drug systems, their cell death pathways, and mechanisms of action in TPDT.

Inorganic-Organic CSNPs Drug System	Cancer Targeting Method	Cell Line	Cell Death Pathway	Mechanism of Action	Ref.
Au@PSMA@MB	Passive targeting (EPR)	Cervical cancer (Hela)	Apoptosis	Mitochondrial destruction	[29]
SiO_2_@CaP@PEG-ZnPc	Passive targeting	Cervical carcinoma (Hela)	Necrosis	Lysosomal disruption	[101]
Fe_2_O_3_@Au-PEG-FA	Active (Folic-Folate receptor)	Nasopharyngeal (KB) and Breast (MCF-7)	Apoptosis	Cell death receptors	[81]
Au@GON-PEG-ZnPc	Passive targeting	Cervical cancer (Hela)	Necrosis and Autophagy	Oxidative stress	[100]
AuNR@SiO_2_ (PEG)-D-CPP-Ce6	Active (Peptide-lipid-targeting)	Breast (MCF-7)	Apoptosis	Activation of death receptor enzymes (caspase 8 and effector caspase 3/7), Mitochondria destruction and ROS generation	[86]
Fe_2_O_3_@C_60_-PEG/HMME	Passive targeting	Mice melanoma (B16-F10)	Apoptosis and Necrosis	-	[85]
ZnO@Au@PEG	Passive targeting	Leishmaniasis (skin cancer mimic)	-	-	[77]
AuNR@PSMA-ICG	Active targeting (Anti-EGFR monoclonal antibody–EGFR binding)	Lung cancer (A549) and Human Keratinocyte nonmalignant cell line (HaCaT)	Apoptosis	Nuclear cleavage of DNA	[76]
Fe_3_O_4_@mSiO_2_@lipid-PEG-ZnPc-methotrexate nanoparticle	Passive and Active targeting	Cervical cancer (Hela) and Lung cancer (A549)	Necrosis	ROS induced membrane oxidation and perturbation.	[96]
GON@PEG-Ce6/Dox	Passive targeting	Cellosaurus cell line (SCC) and SCC bearing mice	-	-	[102]
PEG-PEI@SiO_2_/ZnPc	Passive targeting	Mouse ascitic hepatoma cell line (H22)	Necrosis	Lysosomal and cytosol damage	[97]
Au@PEG-ZnPc/anti-HER2	Active (antibody-antigen targeting)	Breast carcinoma cell lines (SK-BR-3, MDA-MB-231, ATCC and MCF-10A)	Apoptosis	Mitochondrial and increase in caspase -3/7	[46]

EPR (Enhanced permeability retention), PEG (Polyethylene glycol), PSMA (poly styrene-*alt*-maleic acid), CaP (calcium phosphate), MB (Methylene blue), ZnPc (zinc (II) phthalocyanines), FA (Folic acid), GON (Graphene oxide nanoparticles), AnNR (gold nanorods), CPP (cell penetrating peptides), Ce6 (chlorine e6), HMME (Hematoporphyrin monomethyl ether), ICG (indocyanine green), Dox (Doxorubicin), PEI (polyethylenimine). Dash signs (-) indicate cell death pathway or mechanism of action as not studied or defined by authors.

## 6. Conclusion and Future Perspectives

### 6.1. Conclusions

Nanotechnology has advanced significantly in the field of drug delivery, imaging, and therapeutic applications, such as TPDT, photothermal therapy, magnetic resonance imaging, radiation therapy, and chemotherapy. Hybrid inorganic-organic CSNPs are promising candidates for the efficient loading and delivery of therapeutic agents. By using polymers, such as PEG, PLGA, and PSMA, as an organic shell on metal cores, such as gold, silver, iron, and silica, many physiological and biological barriers are easily reduced due to the submicron sizes, enhanced solubility, bio-functionality, and colloidal stability of the hybrid CSNPs. Most importantly, the absorption sites on the organic polymer shell make it easy to attach PS agents and cancer specific ligands for TPDT through EPR (passive targeting) and binding onto receptors overexpressed on the cancer cell surface. Another advantage of hybrid inorganic-organic nanodrug systems is that they are able to be designed with specificity to localize within certain cellular compartments, such as the mitochondria, lysosomes, cytosol, or cytoplasm, which can allow for efficient cell death through the disruption of intracellular organelles. Additionally, the metal core of the hybrid CSNPs has been demonstrated to increase the generation of cytotoxic singlet oxygen, thus improving the efficacy of the treatment through increased oxidative stress on cancer cells. 

### 6.2. Future Perspectives

Despite the promising results demonstrated by hybrid nanodrug systems in TPDT, reproducibility, loading amounts, and defined linking between the polymer functional groups with therapeutic agents is still a challenge that needs to be addressed during the design stages of the hybrid nanodrug system. It is also important to design hybrid CSNPs that are capable of being eliminated from the biological system, post treatment. Asymmetric modification of the organic polymer shell can be used as a form of defined attachment of therapeutic agents, while metal oxides with biological functions, such as iron oxide and zinc oxide, can be used as the core metal. Their degradation post treatment when formulated with biodegradable polymers will be efficient in determining the fate of hybrid nanodrugs after treatment.

## Data Availability

No data available.
